# Supplementary material for: Optimization of the plasmonic properties of titanium nitride films sputtered at room temperature through microstructure and thickness control
Source: Sci Rep. 2024 Mar 8;14:5762. doi: 10.1038/s41598-024-56406-6 (PMC10923920; doi:10.1038/s41598-024-56406-6)
Supplement: Supplementary file 1 — Supplementary Information. [file 41598_2024_56406_MOESM1_ESM.docx]

**Supplementary Information: Optimization of the plasmonic properties of titanium nitride films sputtered at room temperature through microstructure and thickness control**

Mateusz Nieborek^1^, Cezariusz Jastrzębski^2^, Tomasz Płociński^3^, Piotr Wróbel^4^_,_ Aleksandra Seweryn^5^, and Jarosław Judek^1,*^

^1^ Institute of Microelectronics and Optoelectronics, Warsaw University of Technology, Koszykowa 75, 00-662 Warsaw, Poland.

^2^ Faculty of Physics, Warsaw University of Technology, Koszykowa 75, 00-662 Warsaw, Poland.

^3^ Faculty of Materials Science and Engineering, Warsaw University of Technology, Wołoska 141, 02-507, Warsaw, Poland.

^4^ Faculty of Physics, University of Warsaw, Pasteura 5, 02-093 Warsaw, Poland.

^5^ Institute of Physics, Polish Academy of Sciences, Aleja Lotników 32/46, 02-668 Warsaw, Poland.

^*^ Corresponding author, e-mail address: jaroslaw.judek@pw.edu.pl.

**Spectroscopic ellipsometry**

In our study, the structural and optical properties of TiN*_x_* samples were analyzed as a function of N_2_ and Ar flow as well as the deposition time. The thickness values of the analyzed in our work films range from 75 nm to 3270 nm, whereas the surface roughness ranges from 0.58 nm to 16.8 nm. Since ellipsometry is an extremely sensitive technique that can detect layers of thickness down to 0.1 $\dot{A}$ [1] a question arises about the proper optical model to transform the experimentally obtained *Ψ* and *Δ* values to the real and imaginary part of the permittivity. The simplest model assumes that the considered layer is semi-infinite and flat. In such a case, one can use the following transformation to get the pseudo-dielectric function as follows:

$\left\langle\varepsilon\right\rangle=\sin^{2} \theta\left[ 1+\tan^{2} \theta\left( \frac{1-\rho}{1+\rho} \right)^{2} \right]$, (1)

where $\rho=\tan\Psi e^{i\Delta}$. The first hesitation regards whether the examined films are absorbing enough that one can neglect the underneath layers, i.e., TiO*_x_* interlayer and silicon substrate with native silicon oxide layer. Whereas the answer to this question for the thickest films is positive, it turns out that for the thinnest samples, the inclusion of the physically existing layers below the examined TiN film to the optical model may contribute to the result. The second concern is related to surface roughness. This question is often neglected in the literature. Whereas for the flattest samples characterized by the RMS value below 1 nm, it may turn out that this approach is often sensible, the neglection of the surface topography for roughness value above at least a few nm, which, e.g., manifests itself during the ellipsometric measurement as a diffuse reflection, might be concerning.

To address the above-described two doubts, we performed a simple numerical experiment in which we used five optical models to transform the *Ψ* and *Δ* values, which are raw measurement data from the ellipsometric measurement, to the real and imaginary parts of the permittivity. These optical models are 1) the pseudo-dielectric function approach, 2) the multi-layer model with flat surfaces, and the multi-layer model with a rough surface, where the surface roughness is modeled as an effective layer consisting in 50 % of air voids and underlying material which optical constants are calculated with the use of Bruggeman Effective Medium Approximation and characterized by the thickness value that: 3) equals the RMS value; 4) equals 1.5·RMS + 4 Å [2]; and 5) is a fitting parameter.

Figures S1 and S2 illustrate the influence of the optical model details on the extracted results. Figure S1 is related to three samples with different thicknesses deposited with 10 Ar flow, which ensures the least rough surface, whereas Figure S2 is related to three samples with different thicknesses deposited with 100 Ar flow, which leads to the most rough surface.

A comparison of those five models shows a minor influence of the underneath layers and a non-negligible influence of the roughness layer on the extracted material dielectric function. For the first series of samples, the first and second optical models give almost identical results, whereas the fifth optical model seems to stand out from the rest. In the infrared region, the real part of the permittivity takes the least negative values for the first two models and the most negative values for the fifth model. Similarly, the imaginary part of the permittivity takes the least positive values for the first two models and the most positive values for the fifth model. As a consequence, the plasmonic Figure of Merit in the case of the first two models is the highest. The inclusion of roughness leads to a slight decrease in the value of FoM and a decrease in the wavelength at which the FoM takes maximal value. For the second series of samples, particularly for the thickest sample, the inclusion of surface roughness leads to a drastic dependency of the extracted permittivity and related Figure of Merit on the selected optical model, as illustrated in Figures S2c, f, and i. But in this case, contrary to the previously analyzed series of samples, despite the real part of the permittivity being the least negative and the imaginary part of the permittivity being the least positive for the first two models, the inclusion of the surface roughness surprisingly leads to a increase in the maximal FoM value.

The proper choice of the most appropriate optical model for our samples characterized by the finite, non-negligible surface roughness is thus a difficult task. Moreover, the ellipsometric curves do not possess distinctive features that might help impose constraints on the model, and the thickness of the characterized layers prevents additional transmission measurements or usage of the interference approach [3]. To address this problem, we performed the Mean Squared Error (MSE) analysis, which results are presented in Table S1. As can be seen, the fifth optical model, i.e., the model in which the thickness of the additional, most on-top effective layer simulating surface roughness is a variable in the fitting procedure, seems to give the lowest value, suggesting it is the most accurate. And the one we are using in this paper.





**Figure S1.** (a)-(c) Real *ε*_1_ part of the dielectric function, (d)-(f) imaginary *ε*_2_ part of the dielectric function, and (g)-(i) plasmonic Figure of Merit (-*ε*_1_/*ε*_2_) as a function of the wavelength for three stoichiometric samples obtained for 10 sccm Ar flow with a thickness of 145 nm, 498 nm, and 2180 nm. Different curves represent five optical models ("om") to transform the experimentally obtained raw *Ψ* and *Δ* data.





**Figure S2.** (a)-(c) Real *ε*_1_ part of the dielectric function, (d)-(f) imaginary *ε*_2_ part of the dielectric function, and (g)-(i) plasmonic Figure of Merit (-*ε*_1_/*ε*_2_) as a function of the wavelength for three stoichiometric samples deposited with 100 sccm Ar flow with a thickness of 218 nm, 498 nm, and 3270 nm. Different curves represent five optical models ("om") to transform the experimentally obtained raw *Ψ* and *Δ* data.

| sample | MSE values for different optical models | | | |
| --- | --- | --- | --- | --- |
|  | #2 | #3 | #4 | #5 |
| Ar 10 sccm, thickness 145 nm, roughness 0.58 nm | 1.16 | 1.09 | 0.99 | 0.73 |
| Ar 10 sccm, thickness 498 nm, roughness 0.96 nm | 1.57 | 1.36 | 1.20 | 0.82 |
| Ar 10 sccm, thickness 2180 nm, roughness 3.10 nm | 1.93 | 1.20 | 1.01 | 1.00 |
| Ar 100 sccm, thickness 218 nm, roughness 1.96 nm | 3.59 | 3.56 | 3.63 | 3.59 |
| Ar 100 sccm, thickness 747 nm, roughness 2.87 nm | 3.87 | 3.80 | 3.78 | 3.79 |
| Ar 100 sccm, thickness 3270 nm, roughness 16.8 nm | 5.47 | 5.54 | 7.12 | 5.15 |

**Table S1.** Comparison of Mean Squared Error of fitting roughness-dependent ellipsometric models.

**4. Literature**

[1] H. Fujiwara, Spectroscopic Ellipsometry: Principles and Applications (Wiley, West Sussex, UK, 2007).

[2] H. Fujiwara, J. Koh, P.I. Rovira, R.W. Collins, Assessment of effective-medium theories in the analysis of nucleation and microscopic surface roughness evolution for semiconductor thin films, Phys. Rev. B 61, 10832 (2000).

[3] J. N. Hilfiker, N. Singh, T. Tiwald, D. Convey, S. M. Smith, J. H. Baker, H. G. Tompkins, Survey of methods to characterize thin absorbing films with Spectroscopic Ellipsometry. Thin Solid Films, 516, 7979−7989 (2008).
